# A formative study of the sociocultural influences on dietary behaviours during pregnancy in rural Bangladesh

**DOI:** 10.1111/mcn.13713

**Published:** 2024-08-30

**Authors:** Nazrana Khaled, Anna Kalbarczyk, Eleonor Zavala, Atiya Rahman, Mary de Boer, Barnali Chakraborty, Hafizur Rahman, Hasmot Ali, Rezwanul Haque, Kaniz Ayesha, Towfida J. Siddiqua, Kaosar Afsana, Parul Christian, Andrew L. Thorne‐Lyman

**Affiliations:** ^1^ Humanitarian Hub BRAC James P Grant School of Public Health, BRAC University Dhaka Bangladesh; ^2^ Department of International Health Johns Hopkins Bloomberg School of Public Health Baltimore Maryland USA; ^3^ The JiVitA Project, Johns Hopkins Bloomberg School of Public Health Rangpur Bangladesh

**Keywords:** balanced energy protein, Bangladesh, beliefs, food supplement, micronutrients, pregnancy diet, qualitative

## Abstract

Balanced energy protein supplementation (BEP) is recommended for contexts of high maternal undernutrition by the World Health Organization. Despite recent improvements in undernutrition, Bangladesh remains a context where BEP could help accelerate progress towards nutrition goals. In preparation for an effective trial testing a fortified BEP, a qualitative study was undertaken to better understand sociocultural factors influencing dietary behaviours in pregnancy. Married women of reproductive age (*n* = 23), their husbands (*n* = 6) and mothers‐in‐law (*n* = 6) were interviewed, and focus group discussions were conducted with women (*n* = 4). Women had a clear understanding of which nutritious foods are important to consume during pregnancy, including green leafy vegetables, dairy and other animal‐source foods. Many explained affordability as a barrier to consuming those foods with the desired frequency. Women acquired information about diet and nutrition in pregnancy from community health workers as well as other women in the community. Most preferred to seek information from their own networks before formal health care providers. Women and husbands generally had positive views about micronutrient supplements, although some mothers‐in‐law were more hesitant. Some food taboos relating to the consumption of certain foods like duck and pigeon meat persist, mainly stemming from concerns for the unborn child. Opportunities exist to build on existing perceptions of healthy diets, potentially framing food or nutrient supplements as a beneficial ‘add‐on’ to promote a healthy pregnancy. There is a scope to strengthen nutrition counselling, especially for the family members, to dispel myths and misconceptions and promote dietary and other support for pregnant women.

## Key messages

- Women in this part of rural Bangladesh understood which foods were beneficial for health and nutrition during pregnancy and the importance of increasing food intake during pregnancy.
- Women and husbands were more likely to view nutrition supplements as beneficial during pregnancy; some mothers‐in‐law expressed reservations about supplements.
- In the context of a planned effectiveness trial of balanced‐energy and protein supplementation, nutrition education should reinforce existing concepts of the importance of healthy diets during pregnancy and build on them to promote the use of nutritious supplements to meet nutrient needs in pregnancy.
- Financial constraints limit access to nutritious food and micronutrient supplements in pregnancy despite widespread beliefs about their value.

## Graphical abstract

This formative research study from rural Bangladesh explored factors influencing dietary behaviours during pregnancy. Women generally understood the importance of consuming nutritious foods during pregnancy, although many felt they were too expensive to regularly afford. Micronutrient supplements were viewed positively. Findings will inform a trial of balanced protein energy supplementation.

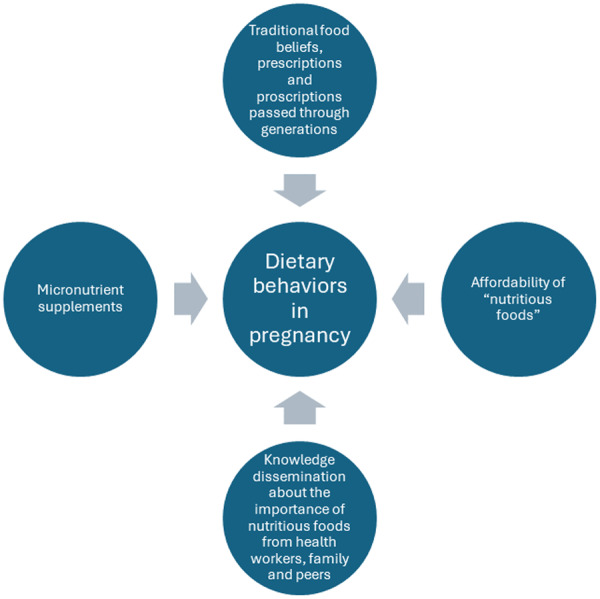

## 1 INTRODUCTION

The food system in Bangladesh has gone through many changes over the past two decades. Increased income, combined with industrialization and globalization of the food system, are driving a nutrition transition in Bangladesh that includes greater consumption of processed foods and animal‐source foods (Al Hasan et al., 2019; Hashan et al., 2020; Hoque et al., 2015). However, even though there is a broader trend of incorporating larger quantities of nonstarch‐based foods into the diet nationally, dietary diversity remains limited (Banik & Rahman, 2018; Gupta et al., 2022; Waid et al., 2018).

Undernutrition and dietary inadequacies persist during pregnancy despite economic development, with important ramifications for both maternal and child health. Nationally, the proportion of ever‐married women with low body mass index (BMI < 18.5) during 2017–2018 was 11.9%, and the prevalence of anaemia among reproductive‐age women in 2011–2012 was 42.4% (National Institute of Population Research and Training [NIPORT], Mitra and Associates/Bangladesh, & ICF International, 2013). Poverty is a strong predictor of women's undernutrition: those in the lowest quintile of socioeconomic status (SES) were five times more likely to be underweight compared to the highest quintile (NIPORT, Ministry of Health and Family Welfare, & ICF, 2020). Low BMI entering pregnancy is a major risk factor for adverse birth outcomes, including small‐for‐gestational age and low birthweight or being born <2500 g (Han et al., 2011). These indicators of poor intrauterine growth are strongly associated with stunting in childhood (Christian et al., 2016; Danaei et al., 2016).

Addressing nutritional deficiencies is an important opportunity to enhance progress towards national targets. The World Health Organization (WHO) recommends iron–folic acid supplementation and nutrition counselling as universal interventions and calcium supplementation, multiple micronutrient supplementation (MMS) and balanced energy protein supplementation (BEP) as interventions that can be considered in certain contexts (WHO, 2016, 2020). MMS trials have shown a significant effect on improving birth outcomes; the largest trial was conducted in Northwest Bangladesh, a setting where 45% of births fell below 2500 g, and showed a reduction in low birthweight by 12% and preterm by 15% compared with iron–folic acid supplementation (Haider & Bhutta, 2017; West et al., 2014). Bangladesh is currently piloting MMS supplementation. While the country has historical experience delivering locally produced BEP in pregnancy through the Bangladesh Integrated Nutrition Project (BINP) from 1995 to 2002 (WHO, 2013), BEP supplements are not currently provided through programmes except in the Rohingya refugee camps (Nahar et al., 2009).

Changes in the food system over time, along with other shifts, such as rising rates of education, have important implications for women's diets in pregnancy and for nutrition interventions focused on pregnancy, such as BEP and MMS, yet few contemporary studies from Bangladesh and the region have explored women's perceptions about what should be eaten during this critical life stage or where they get information from (Nath et al., 2019; Shamim et al., 2016). A historical body of literature on dietary beliefs during pregnancy from South Asia focused on the importance of beliefs about the hot and cold properties of certain foods, prescriptions and proscriptions related to the effects of specific foods in pregnancy, and the phenomenon of ‘eating down’ in pregnancy to prevent the development of a larger baby (Harding et al., 2017; Nag, 1994). However, few recent qualitative studies exist studying dietary behaviours and beliefs in Bangladesh. One recent study examined programmatic constraints related to delivering effective interventions related to maternal nutrition (Rasul et al., 2023), a topic that has also been explored through recent quantitative studies (Kraemer et al., 2023; Nguyen et al., 2017). Another formative research study used focus group discussions (FGDs), qualitative interviews of pregnant women and health care providers and market observations to identify opportunities to inform the design of a pilot study of MMS in two parts of Bangladesh (Sarker et al., 2021).

This qualitative study uses a broad lens to examine the factors that currently influence dietary behaviours during pregnancy in rural Bangladesh, with the main aim of informing the design of a BEP supplementation trial during pregnancy.

## 2 METHODS

### 2.1 Study design and setting

A qualitative formative study was designed to understand sociocultural factors that influence dietary behaviours during pregnancy among women of reproductive age in three unions of northwestern Gaibandha, Bangladesh (Bamandanga, Sonarai, Sarbananda), a subset of areas within the larger JiVitA research site, which has been home to maternal and child nutrition and health research trials over the past 20 years (Christian et al., 2016; Klemm et al., 2008; West et al., 2014).

### 2.2 Participants and sampling

Given that the target group for the supplementation trial was women with a higher probability of becoming pregnant, we focused the formative study primarily on married women 18–35 years of age, referred to in this manuscript as married women of reproductive age (MWRA). The study recruited married women of this age range, their husbands and mothers‐in‐law residing in the three unions.

Our project site maintains a database of all households in the study area. In preparation for this study, Community Health Research Workers (CHRWs) visited their communities to update this database, compiling a list of married women who were pregnant or who had a recent birth in their respective communities. CHRWs visited women's homes, read a general recruitment script and provided information about the study aims. Screening questions were then asked to obtain demographic information on age, parity, education, and SES, and informed consent was obtained from all study participants. A purposive, criteria‐based selection technique was applied to develop a sampling frame to maximize variation in parity, age and SES of participants across the three unions (Douglas, 2022).

After samples were drawn from the compiled list of married pregnant or recently delivered women mentioned above, consenting women were enroled in the study during a second visit by trained interviewers; a subset of husbands and mothers‐in‐law of enroled women were also recruited based on availability. Local antenatal and reproductive health care providers were also purposively selected through local government health centres in all three unions with permission from the Ministry of Health and Family Welfare. All participants were administered an oral consent script.

In‐depth interviews (IDIs) and FGDs, were used to understand the perceptions of the women, husbands, and mothers‐in‐law regarding food, nutrition, information seeking, during pregnancy. Five FGDs, with 4–6 participants, each were conducted with women to understand their attitudes and perceptions towards food consumption and supplement use in pregnancy, and to assess the acceptability and feasibility of BEP supplementation implementation. These numbers were predetermined with the goal of reaching data saturation.

### 2.3 Study tools and data collection

Separate IDI guides were developed for women, husbands and mothers‐in‐law to elicit each group's perceptions and beliefs, guided by aspects of the cultural‐ecological model of food and nutrition (Jerome et al., 1980; Pelto & Armar‐Klemesu, 2015). We explored perceptions of food choices in pregnancy, community and social supports and women's mobility and agency (an intersection of physical and social environment and culture). We did not ask questions specifically about technology but we did code for this in the analysis.

FGD field guides were created to explore themes including women's dietary preferences, perceptions of beneficial and harmful foods during pregnancy, perceptions of micronutrient or packaged food supplements and weight gain during pregnancy. All four field guides (three IDI and one FGD) were piloted in an adjacent area to the study site before data collection.

The qualitative research team consisted of experts and senior researchers from the collaborating institutions with doctorate or master's level education in the areas of anthropology, nutrition, public health, and medicine; all had been trained in qualitative methods. Three qualitative interviewers (QIs), graduates of anthropology, one male and two females, conducted all IDIs and moderated and facilitated FGDs. They were trained by the BRAC University research team (A. R., N. K., B. C.) to collect qualitative data in Bengali.

Data were collected between January and March 2022. Local female community health workers (CHWs) compiled a list of women, who they then contacted via telephone calls or home visits. Participants who were interested were added to a recruitment form, and a suitable time for a visit by QIs was identified. QIs built rapport by calling participants on the phone before visiting their homes and asking about their current situation and daily life. The IDIs with women, their husbands, and their mothers‐in‐law were conducted in their homes. Each interview was 30–60 min in length and audio‐recorded using a tablet. Interview notes were taken using the Livescribe™ Symphony smart pen (Livescribe) linked to tablets to sync audio and notes. FGDs were approximately 60–90 min in duration. During the FGD, one QI acted as moderator and another as note‐taker. At the end of each day of data collection, debrief sessions were held between QIs and supervisors to identify and mitigate challenges, discuss preliminary findings, and adjust questioning strategies.

### 2.4 Data cleaning and analysis

Verbatim transcripts of recorded interviews were prepared in Bengali by each QI and reviewed by two supervisors (A. R., N. K.) fluent in Bengali against audio recordings for completeness and accuracy. Once finalized, the transcripts were translated by three professional translators to English and again reviewed and cross‐checked against Bengali transcripts by the same supervisors to ensure meaning retention, grammatical accuracy and completeness.

A team of six investigators (N. K., A. L. T.‐L., E. Z., A. R., A. K., B. C.) were involved in the analysis. The initial code list was informed by two analytical frameworks. The implementation science in nutrition framework (Tumilowicz et al., 2019) established five domains of interest, and the cultural‐ecological model of food and nutrition (Jerome et al., 1980; Pelto & Armar‐Klemesu, 2015) helped predefine codes related to perceptions of food choices in pregnancy, community and social supports, women's mobility and agency (an intersection of physical and social environment, and culture), and technology Data were coded and managed on the Dedoose software (www.dedoose.com). After 10% of the translated transcripts were initially coded, we refined the code list to capture emerging themes from the initial findings, and the remaining transcripts were then coded. Group analysis meetings were held every week to achieve consensus among investigators and identify emerging themes and concepts; intercoder reliability tests were conducted at the start of coding. Intercoder reliability was tested using Cohen's *κ* Statistics scores on Dedoose. Two senior researchers, experts in qualitative research were assigned as the gold standard and coded sample transcripts. The rest of the team then coded the same transcripts to compare the intercoder reliability. This was done twice to ensure consensus. In this manuscript, we specifically explored themes emerging from the following codes: Pregnancy Diet (Amount and/or Frequency, Food Classification, Micronutrient Supplements, Special Snacks/Foods), Social and Cultural Beliefs and Norms, Information Sources, Households (Access to Resources, Family Support).

### 2.5 Ethical considerations

Ethical approval for this study was obtained from the Institutional Review Boards of Johns Hopkins University and the BRAC James P Grant School of Public Health, BRAC University. Verbal consent was obtained by explaining research objectives, how the information would be utilized, privacy and anonymity, and why it was important or relevant for the participant to take part in the study. The Qis conducted the IDIs in the privacy of homes of the women and FGDs in JiVitA field offices, respectively, following informed consent procedures.

## 3 RESULTS

### 3.1 Participant characteristics

Interviews and discussions were conducted with the MWRAs, their husbands and mothers‐in‐law regarding food and nutrition during pregnancy. Of the 23 women participating in IDIs, most were between 20 and 30 years old, belonged to a low‐to‐medium socioeconomic background and reported being literate, although very few women had a formal education beyond primary school (Table 1). There was an even distribution across parity and pregnancy status. Husbands were between 21 and 40 years old, and most reported being literate. Mothers‐in‐law were all older than 40.

**Table 1:** Sociodemographic characteristics of interview participants.

**Respondent type**	**MWRA (*n*** = **23)**	**Husband (*n*** = **6)**	**MIL (*n*** = **6)**
Age (y)			
15−20	8	–	–
21–30	12	4	–
31–40	3	2	1
>40	–	–	4
Uncertain			1
SES			
Low	9	–	–
Medium	10	3	3
High	4	3	3
Literacy			
Illiterate	3	1	2
Literate	20	5	4
Parity			
No children	8		
Primiparous	9		
Multiparous	6		
Pregnancy status			
Nonpregnant/lactating	7		
Pregnant	9		
Lactating	7		

Abbreviations: MIL, mothers‐in‐law; MWRA, married women of reproductive age; SES, socioeconomic status.

FGD participants were women between 18 and 35 years of age and varied in age, parity and education level. FGD women were mostly literate with the exception of a few participants, yet education levels varied between grade 2 to even master's level education for some women, although this was very rare. Most of the women had primary‐level education (class 2–5) and worked at home.

### 3.2 Findings

The presentation of findings from this research is structured by six major themes: (i) Food consumed and avoided during pregnancy, (ii) Amount and frequency of food intake during pregnancy, (iii) Perceptions of micronutrient supplements, (iv) Perceptions of special packaged food supplements, (v) Sources of information about what to eat during pregnancy and (vi) Family support and access to resources.

#### 3.2.1 Food consumed and avoided during pregnancy

When asked whether there were specific types of food that women should eat during pregnancy or for a healthy pregnancy, most women and their family members described the importance of consuming ‘pushtikor’ (nutritious) foods. These foods were described as being good for the body and included dairy, eggs, meat, fruits and vegetables. Almost all respondents mentioned three or more of these food categories. Some participants specifically mentioned eggs from *desi* (local) chicken breeds. Some mentioned certain types of small fish and fruits, such as apples, oranges, papayas and bananas, as well as meat (chicken, beef or goat).

One needs to eat a lot of things, ‘pushti‐tushti’ (nutritious food) (during pregnancy). We are poor, we can't procure those food items. Milk, egg, meat, vegetables, ‘mishti kumra’ (sweet pumpkin), ‘pepe’ (papaya), these should be eaten more. Before, I used to eat less of these. It is good to drink milk, eat eggs, any fruit, vegetables, ‘chotomaach’ (small fish), these are good foods. Eating these things is good for nutrition; the baby will be well and I will also be well. (Woman, age 33, parity 1)

Eggs and milk were frequently described as necessary to eat daily during pregnancy, and vegetables were mentioned as a good source of iron and calcium. One participant described the importance of variety and appetite for being able to eat well:

A pregnant woman can eat different types of food on different days… If one has appetite, they are able to eat well, or else even delicious food does not taste well. (Woman, age 38, parity 3)

There was a common understanding that whatever the mother eats affects the baby; hence, if the mother eats nutritious food, the baby will benefit as well.

The baby's bones will be good; its body structure will be good. If they do not eat, then their baby might lose weight (‘baccha shuki jay’). The child is becoming ‘protibondhi’ (disabled). Eating nutritious food means keeping the baby healthy and helping it to grow healthy and strong. (Woman, FGD 01)

Despite wide recognition of the importance of nutritious foods, the ability to afford such foods was described as a major constraint to their consumption, particularly for—more expensive items such as meat (chicken, beef and mutton). In comparison, fruits and vegetables were viewed as more affordable. As noted by a husband:

The fact is that we all are needy people, we provide (pregnant mothers) with the food that we can afford. (Husband, age 40)

#### 3.2.2 Food proscriptions

A variety of foods were reported to be avoided by women during pregnancy. Common reasons included beliefs that the food could be harmful to the baby, physical properties of food (e.g., one should not eat slippery foods), and beliefs that the food could cause a cold or excess gas and discomfort to the mother.

Many beliefs were related to the mother's diet having an effect on the baby. Frequently mentioned foods to avoid included taro roots (*kochur mukhi*), duck meat (*hasher mangsho*), duck eggs (*hasher dim*), pigeon meat (*kabutar er mangsho*) and shrimp *(chingri)*, as these were believed to cause breathing problems in the baby. Occasionally, participants brought up other anecdotal traditional beliefs about the influence of certain food items on birth or health outcomes. Even though participants often questioned the validity of some of these beliefs, at the same time, they suggested that they could influence the intake of such foods:

According to my understanding, there aren't any foods that can harm a pregnant woman, but I am not feeding my wife fish. She does eat fish, but very selectively. I am feeding her desi fish (local fish). She can eat Rui mach (*Labeo rohita*), Bata mach (*Labeo bata*), choto mach (small fry), puti mach (small fry) at the moment. She is not eating Mirka fish (mrigal carp), that is, the fishes which have bigger heads, she doesn't eat those ones. This is because people say abol tabol (ridiculous things), you know? If you eat fish with bigger heads, then the baby's mouth will be as big as the fish head etc. (Husband, age 23)

Certain types of fruits such as pineapple, papaya, grapefruit, guava and watermelon were commonly avoided due to a belief that these could cause the baby to become ‘noshto’ (miscarried/aborted), cause wounds in the baby's head, or cause epilepsy:

Doctors say not to eat these types of foods. Eating these foods will cause harm to the baby. Their skin will peel off. The baby may become ‘noshto’ (miscarried). You are prohibited to eat these things within three months of the pregnancy; if you do, the baby will become miscarried. The community health worker apa has also said these things. (Woman, age 17, parity 1)

However, both pineapple and papaya were also noted by some people as foods that would be good to consume during pregnancy; one woman noted the inconsistency that although some health care workers said to avoid them due to the possibility that they could cause miscarriage, the posters hung in the health care centres encouraged more consumption during pregnancy.

Some women tended to avoid foods that smelled bad during pregnancy, even things they normally consumed. This included items they considered to be nutritious, such as milk and eggs. Women also avoided deep‐fried food, spicy or oily food, as well as stale food as these types of food cause gastrointestinal problems and discomfort. Sour food was avoided as it was thought to cause ‘blood bhanga’ (bleeding) and might cause the pregnant woman's water to break.

I cannot eat oily and ‘bhaja‐pora’ (deep‐fried) food as it causes me problems. For example, I cannot eat spicy food, deep‐fried food, and of course, one should not eat stale foods. Spoiled food that has become sour and smelly should not be eaten as it harms even normal and healthy people, and, during pregnancy, it is strictly prohibited. I do not know what types of harm it causes but it causes issues. For example, it causes ‘pet kharap’ (stomach upset). (Woman, age 20)

#### 3.2.3 Amount and frequency of food intake during pregnancy

Participants had varying opinions about the amount of food women should eat during pregnancy. Many women were in favour of eating more during pregnancy overall, driven by the belief that increasing the amount of food intake would aid the physical and mental development of the child and the general health and well‐being of the mother.

When a woman becomes pregnant, the food intake of the mother and the child should be increased, and (food eaten) more frequently; it will help the child with physical and mental development. Children of mothers who do not eat adequate and nutritious food will not be healthy and they become weak as they don't have proper brain development. When the mother does not have adequate food, it makes her weak and sick, such as having seizures in the abdomen and arms and legs, headaches, and vomiting. (Woman, FGD 1)

Others felt that eating more would be detrimental to the child's growth in the womb. This belief may stem from the idea that food and the child occupy the same space in the mother's belly, as discussed in one of the FGDs:

(Participant 4) There is a rumor in the village that if I eat too much then the child becomes small in the womb, is that true? I think this concept is right, absolutely right (Participants 2 and 3). The child cannot become large in size if the stomach is always full (Participant 2). (Woman, FGD 1)

To address the issue of limited space, many women preferred eating more frequently than usual but in smaller amounts, such as four times per day instead of three.

She should eat in small amounts more frequently. If she eats rice (in the morning), then at 12, she can eat a biscuit, or some fruits. If she eats these, there won't be too much pressure inside (her stomach). The pregnant woman's demand for food is met and she can remain healthy, too. (Woman, age 38, parity 3)

Mothers‐in‐law also emphasized eating in small portions, more frequently, despite some pregnant women's reluctance to eat due to a lack of appetite. The pregnant women believed eating less would lead to the baby moving around more inside the womb and cause the mother to become sick.

#### 3.2.4 Perceptions of micronutrient supplements

Women and husbands had generally positive opinions about the value of taking micronutrient supplements (tablets) during pregnancy. Many women and husbands understood the importance of such supplements—especially iron, vitamins and calcium—and some women reported taking a combination of these during their pregnancies. They felt that taking such supplements was essential for the health of the mother and the child, as they could prevent malnutrition and develop the child's intelligence.

Yes, it is definitely important (to take iron, calcium, vitamin tablets) because taking medicines is essential during pregnancy. These tablets are beneficial; not having these causes malnutrition. The child needs to be healthy, and for that, the mother needs to take those iron, calcium, and vitamin tablets. These tablets are needed even though we are taking normal foods; these kinds of advice are given by the doctors… everyone takes these (during pregnancy). These are good for both mother and the child. (Woman, age 20)

Other reasons for taking micronutrient supplements included making up for the lack of a specific nutrient in the body, preventing blood deficiency, and relieving bodily aches and pains:

When you take calcium tablets, it helps recover from the lack of calcium in the body. That also helps to strengthen the bones of the child. Many times, the mother is unable to eat food adequately because of the child, which causes aches in the legs and arms. These conditions are healed by the calcium tablets. And Iron tablets recover the mother's blood deficiency. (Woman, FGD 1)

Women mentioned that supplements may be appropriate for mothers who cannot obtain sufficient nutrition from their regular diets due to physical inability (e.g., vomiting or morning sickness). However, supplements were not always affordable, and some women preferred not to take them, as highlighted by this husband:

(My wife) does not take vitamin and iron tablets. There is a reason—(rich people) have money, poor people like us will not eat these, we are afraid, we do not have money; you have to understand (our position)… She (wife) tried a ‘pushti packet’ (nutritious packaged food), but she did not like to eat that. She has no interest or appetite for these types of food. As far as I know, my wife feels healthy. As long as she feels healthy, it's okay. But sometimes the doctor says some bhongchong (something ridiculous) (about taking supplements). I don't believe in the things they say at all. My point is, if I am physically well then (I don't need these things). (Husband, age 23)

One reason for avoiding supplements in pregnancy was due to beliefs that the baby would become too big and a caesarean section would be required. Many of these perceptions were generated by elder members of the community, such as some mothers‐in‐law who expressed a more negative view of nutrient supplements.

(Elders say) now, ‘there are so many tests, so many medicines, still the babies need to be delivered by cesarean section… we didn't take vitamins, still our children were born normally’. (Woman, age 27, parity 2)

#### 3.2.5 Perceptions of special packaged food supplements

Women were asked about whether there were any special foods in a packaged form available for pregnant women. They were also asked about what they perceived the role of such foods could be if they were introduced into a woman's diet during pregnancy. A few recalled hearing about ‘pushti packets’, or nutritious food packets given during the BINP programme implemented in Bangladesh from 1995 to 2002. Some also mentioned ‘Mother's Horlicks’, a product currently available in the market for pregnant women:

For me personally, I feel these things (specially packaged food) are also good for health. If it is beneficial, then it should be eaten. Beneficial in the sense that you can have it if you are getting hungry within an hour, you can have something like this. As far as I know, this type of packet food for the pregnant mother is not available in the market, but I know that there is Mother's Horlicks. Then there are milk and eggs for the mother. (Woman, age 19, parity 2)

The same participant raised cost as a barrier and preference for a ‘normal’ (i.e., home‐cooked) diet among most women.

It will take time for the pregnant women to have it (packet foods). You can buy it as you have money whereas I cannot, as I don't have an income. I think if there was a type of food like this, pregnant mothers would not like it. Pregnant mothers would think, ‘What good will it do to me?’ Pregnant women would rather eat rice, vegetables, fish, more green vegetables, that's what they would think. Some pregnant women will not like it. (Woman, age 19, parity 2)

Most women reported that they ate what was normally cooked at home and eaten by everyone else in the family. Few described anything being prepared especially for them during pregnancy. Some mothers‐in‐law felt that the current generation of pregnant women have more options than they had.

Nowadays previous rules and regulations do not exist, now they eat apple, orange, banana, eat mangoes in season, drink nutritious milk, now I heard that they also drink Horlicks, eat fish, eat meat, boiled eggs… These were not there in my generation; now many things are available… (Mother‐in‐law, age unknown)

#### 3.2.6 Sources of information about what to eat during pregnancy

Women seek and receive information on pregnancy diet from a variety of sources, including their mothers, mothers‐in‐law, sisters‐in‐law, neighbour women, health care providers such as midwives, CHWs and health providers who sit at community clinics and upazila health centres. When women gather, they discuss various topics and problems that they face during pregnancy and give each other advice.

You can't know everything from one person. Normally women sit down and chat, they discuss anyway and give each other advice. (Woman, age 35, parity 2)

Women with prior pregnancy experience and those who work as community health providers or birth attendants were both important sources of information. Women tended to go to doctors specifically when there was a problem, although doctors were frequently mentioned as highly regarded sources of information, providing advice on what foods to eat or avoid during pregnancy as well as prescribing micronutrient supplements, and providing advice on adequate weight gain during checkups. However, the term ‘doctor’ or ‘daktarni’ (female doctor) seemed to be loosely defined and was used both for trained, formal service providers and also health workers who visit door‐to‐door. Health workers who visited door‐to‐door or could be reached over the phone were also consulted for diet‐related advice during pregnancy. Some participants also sought the advice of homoeopathic providers.

Many women reported that they did not have easy access in general to pregnancy‐related information (not specific to diet or nutrition advice), although their husbands or mothers‐in‐law generally felt that was not the case. This contrast is illustrated by the divergent statements of one woman and her mother‐in‐law:

I didn't get the information easily, they said everything when they came (to my home) face‐to‐face. I didn't have everything at hand always, maybe sometimes the community health worker apa was not there. (Woman, age 22, parity 3)

The information that the (pregnant women) need, they are getting those properly. And they don't have any problem, it is easy to get the information. (Mother‐in‐law, age 40)

Some mothers‐in‐law felt that the novelty of first‐time pregnancy and associated shyness in young women deter them from seeking out information initially and that some mothers‐in‐law were not cognizant of where their daughters‐in‐law would go for information.

When a woman becomes pregnant for the first time, she wouldn't know anything. She feels shame to express this to just about anyone; only the besharma (shameless) women talk about it. Out of shame, most women can't tell to anyone. It's a problem if she doesn't tell it to anyone. (Mother‐in‐law, age 66)

I don't know from whom they come to know (pregnancy‐related information). They don't discuss these things with me. If the pregnant women call the community health worker, she comes (and gives information). (Mother‐in‐law, age 66)

Similarly, many husbands were often unaware of the types and sources of information their wives sought. Most husbands seemed to prefer that their wives consult with a doctor or formal health care provider for health‐related advice.

If she wants to take advice, she can take it from the health center, or from the clinic. If her husband goes with her, there is an added benefit, as, even if other people cannot understand (your condition), your husband will understand. (Husband, age 37)

#### 3.2.7 Family support and access to resources

One of the major factors preventing women from eating what they consider to be nutritious food was their economic context. The theme ‘eat as per your ability’ emerged from the data. Family income and support was important in enabling women to access nutritious food:

Rich people will feed (pregnant women in their families) a lot. Suppose ‘ajke je jinish amader naare dhukena’ (directly translated to: the food that we can't eat because it doesn't fit into our intestines. Meaning, the food that they can't digest or don't have the taste for), the rich will feed their families these things. They have to bring it from wherever they can. With the little amount of money I earn, if my wife wants to eat something, I bring it for her myself and feed her. But it is not possible for me to bring it regularly. (Husband, age 23)

Most of the women did not regularly go to the markets to buy food. When they went, they usually had an accompanying person or chaperone. Their husbands and sometimes mothers‐in‐law bought food and other necessary goods. Decisions on which foods to purchase varied from household to household, not always according to the woman's preferences:

I never go outside to purchase important things. I tell my husband or my father, and they bring (whatever I need) from the market. I don't have that type of hobby to go shopping, whatever I need they can manage. I only go there when I truly need to go. My husband or my mother or mother‐in‐law goes along with me. (Woman, age 22, parity 1)

Husbands often expressed the desire to be supportive as well.

I buy food, medicines, whatever she needs from the market. If she needs medicine, then I bring that after consulting with the doctor and purchasing other things from different stores. (Husband, age 28)

Support for chores and caregiving from family members varied. Some husbands were very caring and involved, both in terms of the physical and mental well‐being of their wives. Ways of helping included buying nutritious food from the market, preparing meals and providing reminders to take medicine and food at proper times.

When I was pregnant, in the morning my husband used to take me on a walk. He used to take me on a walk in the morning from there to there (approximately 15‐minute walk), then used to give me two glasses of water, brought me eggs for breakfast in the morning, and often used to give me medicine and one glass of milk with turmeric to drink. (Woman, age 20, parity 1)

However, many husbands also reported having little spare time to help their wives at home.

Yes, (my wife) can't bring heavy things during pregnancy, I bring that or while cooking she needs water, I bring to her if I have time. When I am free, only at that time I help her. I also work, so when I have leisure time only then I help her. I always remind her to eat at the proper time. There are not many household activities, we have a small family, I do the work that she can't do. (Husband, age 28)

Some husbands supported the idea of accompanying wives to their doctor's consultations to directly receive information and recommendations from the doctor.

It's true, if you hear directly, you can easily understand everything rather than from someone else (who accompanied your wife for the checkup). Then, there may be some informational mismatch. That's why it will be better if the husband goes with the women. (Husband, age 37)

Some mothers‐in‐law and other family members helped with cooking and other chores when the woman was pregnant and were positive and supportive of healthy eating during pregnancy:

My mother‐in‐law does everything for me, she never allows me to pull heavy things, never allows me to push the tube‐well… Everyone helps me, my mother‐in‐law helps me. She also does cooking. My family members look after my child, and also look after me, provide me treatment. (Woman, age 22, parity 2)

Other mothers‐in‐law were unable to help due to old age or not fully supportive of women needing rest during pregnancy, and if they helped, they did so begrudgingly.

(She says this with a little resentment) Need to do ‘bari ghorer shamta shora’ (that is, sweep the house), ‘randi‐bari deya’ (doing the cooking). These are the jontrona (hassles) of the wives these days. The wives of previous generations didn't need so many things. The wives these days are ‘thyang alo‐thyang toley tolot’ (that is they just sit there with their legs crossed and eat). Their concept after being pregnant is that, they are pregnant right now, they can't move, they can't cook, then what work can we do for you? So now we need to prepare everything for them. (Mother‐in‐law, age 40)

## 4 DISCUSSION

This research aimed to investigate the current factors influencing the dietary behaviours of women during pregnancy in the rural Northwest region of Bangladesh. Our findings revealed prevalent and consistent beliefs and practices regarding what constitutes ‘nutritious foods’ and the need for the consumption of additional nutritious food in pregnancy to benefit the baby and the mother. We also identified other key factors, including the perceived unaffordability of many nutritious foods and the perceived benefits of micronutrient supplements by women. These findings highlight potential opportunities to promote recommended supplements such as iron–folic acid, MMS and BEP to augment nutritional counselling during pregnancy to improve pregnancy health and birth outcomes (WHO, 2016).

The consumption of additional nutritious food is important during pregnancy to optimize health outcomes (Abu‐Saad & Fraser, 2010; Nnam, 2015). Our findings highlighted how women and families valued the need for higher consumption of nutritious foods during pregnancy. Similar knowledge and awareness have been shown in other studies from Bangladesh (Levay et al., 2013; Sarker et al., 2021). Leafy green vegetables, meat, milk and eggs were among the most nutritious foods for pregnant women identified by participants in our study; many of these were also identified as important for pregnancy in another study conducted in the Southern and Northern districts of Bangladesh (Sarker et al., 2021). It is possible that these foods were consistently identified due to effective nutrition counselling by CHWs (Levay et al., 2013), as many of the items (especially fish, milk, eggs and vegetables) have been included in the Bangladeshi Ministry of Health's nutrition communication materials (Director General Health Services (DGHS) & Ministry of Health and Family Welfare (MOHFW), Government of Bangladesh, 2018). However, in our study, many women also drew attention to the role of informal networks in reinforcing dietary and other pregnancy‐related advice, perhaps suggesting indirect diffusion of nutrition counselling interventions in pregnancy.

Despite the existence of a common understanding of nutritious foods, many of those foods were viewed as unaffordable by much of the population, particularly animal source foods. Similarly, they were familiar with micronutrient supplements and had basic knowledge of the reasons and value of using such supplements during pregnancy. Evidence from other studies has also pointed to the unaffordability of many nutrient‐rich foods by low‐income rural populations in Bangladesh and elsewhere in South Asia (Khatun et al., 2020; Na et al., 2016; Robert et al., 2021; Sufyan et al., 2019; Wallace et al., 2014). Thus, despite a high level of knowledge and awareness about nutritious foods in the population, dietary quality during pregnancy is compromised due to the unaffordability of many of those nutritious foods (Shamim et al., 2016).

These findings have several programmatic implications for potential BEP supplementation in pregnancy. First, communication strategies that highlight the nutritional advantages of fortified food supplements in pregnancy could help to enhance the acceptance of the supplements by women. Second, for many potential consumers, it is likely that the supplements would either need to be distributed for free through a programme or offered at a subsidized cost to ensure affordability. Product pricing will also be a crucial factor to consider if the supplements are made available on the private market.

In addition to cost, sociocultural norms and beliefs and practices related to food in pregnancy influence women's dietary choices and intakes. Many respondents described beliefs that certain types of food, including meat, duck eggs, vegetables and certain fruits, should be avoided during pregnancy because they may cause allergies, colds, or developmental problems in the offspring. Similar beliefs and practices regarding food avoidance have been reported in other parts of Bangladesh (Sarker et al., 2021; Shannon et al., 2008). Our findings that women also avoided fish and meat due to nausea, vomiting or reduced appetite also echo similar findings from other studies in South Asia (Christian et al., 2006; Shannon et al., 2008).

Many previous studies from South Asia have highlighted beliefs about the humoral (hot‐cold) properties of foods as important for pregnancy and lactation (Christian et al., 2006; Harding et al., 2017; Shannon et al., 2008). However, our study participants rarely mentioned this phenomenon, apart from the risk of cold or breathing issues developing in the unborn baby from the mother consuming ‘cold’ food. Additionally, despite the prevalent belief among women participants of the benefits of consuming additional nutritious foods and micronutrient supplements during pregnancy, some mothers‐in‐law expressed concerns about the fear of having a large baby and birth complications. The extent to which mothers‐in‐law can influence actual behaviour related to supplement use or food consumption, however, is uncertain. While previous research from Bangladesh found that fear of large babies and consequent caesarean section could act as a barrier to uptake and adherence to daily supplementation (Alam et al., 2015), our findings suggest the possibility of a generational shift in beliefs, which could be reinforced through continued behaviour change communication interventions.

The long relationship between our project and the local community in Gaibandha led to a strong rapport between interviewing teams and study participants. We were able to reach saturation on many key topics and many of the findings from individual interviews also aligned with those from the FGDs. Another key strength of this research was achieving methodological and data source triangulation, which is viewed as a research strategy that can increase the validity of findings (Carter et al., 2014). However, our study was limited by the smaller number of interviews with husbands and mothers‐in‐law, which restricted our ability to characterize intrahousehold support given the variability in family relationships and power dynamics. For example, while we were able to ascertain the beliefs of mothers‐in‐law, we did not have sufficient information to confirm the extent to which this influenced dietary practices in women during pregnancy. Given the small sample size of husbands and mothers‐in‐law, the generalizability of these findings is uncertain.

### 4.1 Implications for BEP supplementation in Bangladesh

Our study found that most women and their families have knowledge about the importance of nutritious foods and the potential value of micronutrient supplementation during pregnancy. Yet the ability of many women to translate at least some of this knowledge into practice is often limited, particularly due to the perceived high cost of nutritious food items and micronutrient supplements, but also due to limited agency in purchasing decisions. Although social protection programmes, including the maternal health voucher programme, exist, greater efforts to enhance access to micronutrient‐dense foods in pregnancy may be needed, potentially including BEP supplementation.

## AUTHOR CONTRIBUTIONS

Parul Christian, Andrew L. Thorne‐Lyman, Anna Kalbarczyk and Eleonor Zavala designed the study and the protocol. Nazrana Khaled and Atiya Rahman trained the field data collectors. Hafizur Rahman, Hasmot Ali, Kaniz Ayesha, Rezwanul Haque, Towfida J. Siddiqua and Kaosar Afsana coordinated the implementation of the study and field data collection. Eleonor Zavala, Atiya Rahman, Nazrana Khaled, Barnali Chakraborty, Anna Kalbarczyk, Mary de Boer and Andrew L. Thorne‐Lyman analyzed and interpreted the data. Nazrana Khaled and Andrew L. Thorne‐Lyman drafted the manuscript. All authors provided critical feedback and approved the final version.

## CONFLICT OF INTEREST STATEMENT

The authors declare no conflict of interest.

## Data Availability

Data that support the findings of this study are available from the corresponding author upon reasonable request.
